# Myocardial Angiotensin-Converting Enzyme 2 Protein Expression in Ischemic Heart Failure

**DOI:** 10.3390/ijms242417145

**Published:** 2023-12-05

**Authors:** Vitalija Siratavičiūtė, Dalia Pangonytė, Lina Utkienė, Lina Jusienė, Jolanta Marcinkevičienė, Zita Stanionienė, Reda Radikė

**Affiliations:** Laboratory of Cardiac Pathology, Institute of Cardiology, Lithuanian University of Health Sciences, 50161 Kaunas, Lithuania; vitalija.sirataviciute@lsmu.lt (V.S.); lina.utkiene@lsmu.lt (L.U.); linapec@gmail.com (L.J.); jolanta.elena.marcinkeviciene@lsmu.lt (J.M.); zita.stanioniene@lsmu.lt (Z.S.); reda.radike@lsmu.lt (R.R.)

**Keywords:** angiotensin-converting enzyme 2, ischemic heart disease, heart failure, immunohistochemistry

## Abstract

The angiotensin-converting enzyme 2 (ACE2)-angiotensin-(1-7)-Mas receptor axis plays a significant role in regulating myocardial remodeling and the development of heart failure (HF), with ACE2 being the primary focus. However, contemporary understanding of the membrane-bound form of the human ACE2 protein remains insufficient. The purpose of this study was to determine the expression of ACE2 protein in different cells of the left ventricular myocardium in non-diseased hearts and at various stages of ischemic HF. A total of 103 myocardial tissue samples from the left ventricle underwent quantitative and semi-quantitative immunohistochemical analysis. Upon assessing ACE2 immunostaining in all myocardial cells through unselective digital image analysis, there was no change in the stage A HF group. Nevertheless, the expression of ACE2 membrane protein in cardiomyocytes showed a tendency to increase, while non-cardiomyocyte ACE2 expression decreased significantly (*p* < 0.001). In the stage B HF group, the intensity of ACE2 immunostaining continued to increase with rising cardiomyocyte ACE2 expression (*p* < 0.001). Non-cardiomyocyte expression, in contrast, remained similar to that observed in the stage A HF group. In the stages C/D HF group, ACE2 expression reached its highest level in cardiomyocytes (*p* < 0.001), while ACE2 expression in non-cardiomyocytes was the lowest (*p* < 0.001). These changes in ACE2 protein levels are associated with left ventricular remodeling in ischemic HF.

## 1. Introduction

Over the last decade, there has been an increase in the prevalence of heart failure (HF). While urgent revascularization and subsequent treatment strategies have significantly reduced mortality in cases of acute myocardial infarction, chronic ischemic heart disease remains the everlasting cause of HF [1,2].

The angiotensin-converting enzyme (ACE) 2-angiotensin-(1-7)-Mas receptor axis plays a pivotal role in regulating myocardial remodeling and the development of HF, with ACE2 being a central player [3,4,5]. ACE2 is responsible for converting angiotensin I to angiotensin-(1-9) and metabolizing angiotensin II into angiotensin-(1-7). Both angiotensin-(1-7) and angiotensin-(1-9) have demonstrated significant advantages for the cardiovascular system [6,7]. Angiotensin-(1-7) binds to the Mas receptor (MasR), thereby regulating downstream molecular pathways, including the mitogen-activated protein kinase, protein kinase B, and oxidative stress-related pathways [8,9,10]. ACE2 not only suppresses the accumulation of angiotensin II but also downregulates the angiotensin II type 1 receptor (AT_1_R). Consequently, the ACE2-angiotensin-(1-7)-MasR axis exerts an opposing effect compared to the ACE-angiotensin II-AT_1_R axis. The ACE2-angiotensin-(1-7)-MasR axis carries a cardioprotective effect and promotes vasodilation during the remodeling process [4,11].

ACE2 is a type I transmembrane protein, consisting of an extracellular N-terminal domain containing the carboxypeptidase site, heavily N-glycosylated, and a short C-terminal cytoplasmic tail. Two forms of the ACE2 protein exist: the cellular full-length protein, which is membrane-bound, and the circulating (soluble) protein. Full-length ACE2 protein anchored to the cell membrane undergoes cleavage by ADAM17, a disintegrin and metalloproteinase domain-containing protein, and forms a soluble ACE2 form that is subsequently released into the extracellular environment [12]. Recent studies have demonstrated a correlation between the increased plasma activity of soluble ACE2 and the development of adverse cardiac remodeling in patients with cardiovascular diseases and HF. It also serves as a prognostic marker for cardiovascular and all-cause mortality [13,14,15]. Therefore, the ectodomain shedding of ACE2 plays an important role in the cardiac remodeling process [16,17].

However, our understanding of the membrane-bound form of human ACE2 protein remains insufficient. Recent years have witnessed a surge in research on *ACE2* gene expression in multiple organs, including the heart, due to its role as a receptor for SARS-CoV, which is pivotal in the infection caused by SARS-CoV-2 [18,19,20]. Simultaneously, transcriptomic studies employing single-nucleus RNA sequencing have shed light on variations in *ACE2* expression levels in various cell types, such as cardiomyocytes, fibroblasts, endothelial cells, and others, both in non-diseased and HF patient hearts. Nevertheless, these studies have been limited to small cohorts of individuals [21,22,23]. Although some studies have been conducted through immunohistochemical analysis of human heart tissue, they do not provide a comprehensive overview of ACE2 protein expression in myocardial cells in non-diseased heart tissue and HF settings [24,25].

This study aimed at determining ACE2 protein expression in left ventricular myocardial cells at various stages of HF resulting from ischemic heart disease, including stage A (at-risk for HF), stage B (pre-HF), and stages C/D (symptomatic and advanced HF) [26], by quantitative and semi-quantitative immunohistochemical evaluation. Additionally, we sought to explore the correlation between ACE2 protein expression and the marker of remodeling, namely left ventricular mass. These findings, obtained through immunohistochemical techniques, are expected to stimulate further sequencing investigations and will enhance our understanding of ACE2 protein expression in the human heart.

## 2. Results

**Figure 1:**
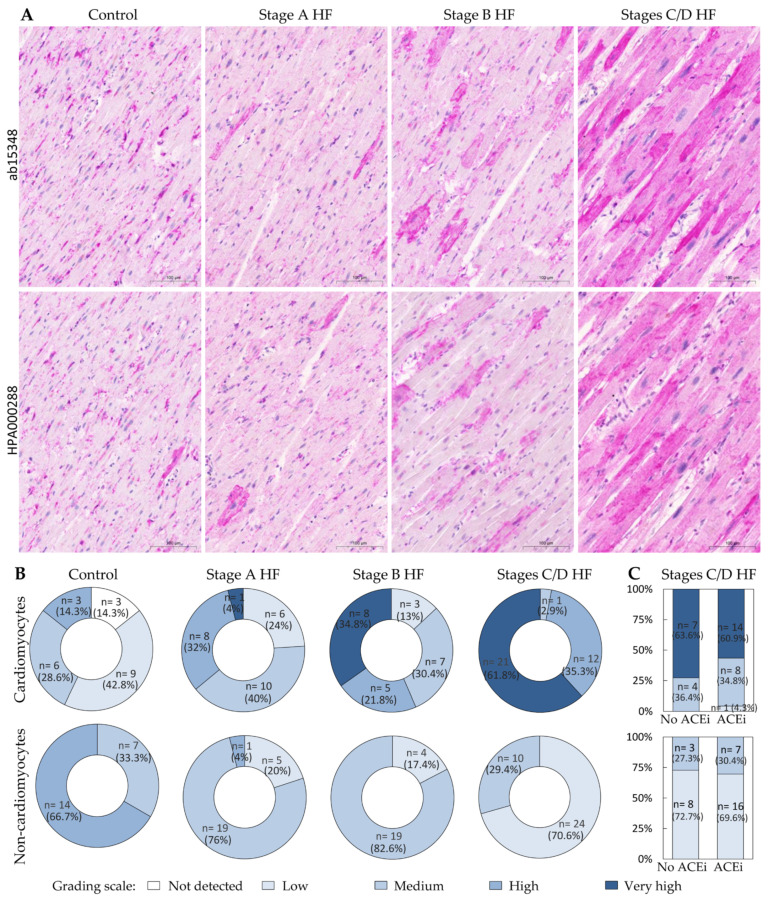
Expression patterns of ACE2 in different groups. (**A**) Representative images of human myocardium immunohistochemistry for both antibodies (stained with magenta, visible in the cellular membrane and cytoplasm). (**B**) Pie charts depict group stratification according to the grading scale. (**C**) The effect of ACEi administration on ACE2 expression. Abbreviations: ACE2, angiotensin-converting enzyme 2; ACEi, angiotensin-converting enzyme inhibitors; HF, heart failure; stages A, B, C, and D HF according to ACC/AHA classification. Scale bar: 100 μm.

To assess ACE2 protein expression within the myocardium in various stages of ischemic HF, a two-antibody study targeting non-overlapping epitopes was performed. Figure 1A shows that ACE2 expression in immunohistochemically stained myocardium was consistent between the antibody targeting amino acids 750 to the C-terminus (ab15348) and the antibody targeting amino acids 1-111 (HPA000288). Immunostaining revealed membranous and cytoplasmic patterns in cardiomyocytes and other myocardial cells. Notably, the antibodies distinguish between ACE2 isoforms: the antibody detecting both the long and short isoforms (also known as delta) spans from amino acids 750 to the C-terminus, while the antibody targeting amino acids 1-111 solely identifies the long isoform. Consequently, the myocardium predominantly expresses the long ACE2 isoform. Subsequently, we conducted quantitative and semi-quantitative analyses utilizing the ACE2 ab15348 antibody against both isoforms to evaluate myocardial immunostaining.

**Figure 2:**
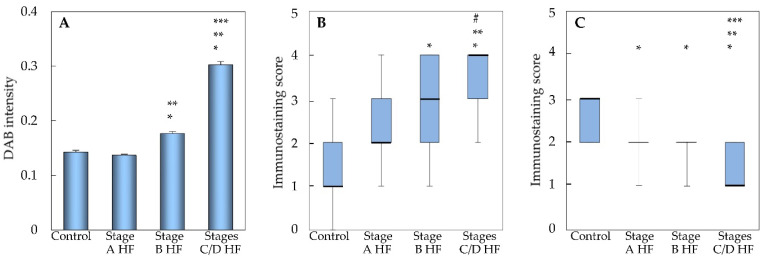
ACE2 expression in myocardium of different groups. DAB stain digital image analysis (**A**), data are presented as mean and standard error; nested ANOVAs with Bonferroni post hoc test were applied. Semi-quantitative immunohistochemical analysis of cardiomyocytes (**B**) and non-cardiomyocytes (**C**); data are presented as median, the 25th and 75th percentiles, and minimum and maximum values (whiskers); the Kruskal–Wallis test with Bonferroni-adjusted pairwise comparisons was used. * *p* < 0.001—stage A HF, stage B HF, stages C/D HF vs. control group data; ** *p* < 0.001—stage B HF and stages C/D HF vs. stage A HF group data; *** *p* < 0.001—stages C/D HF vs. stage B HF group data; # *p* < 0.05—stages C/D HF group vs. stage B HF group data. Abbreviations: HF, heart failure; stages A, B, C, and D HF according to ACC/AHA classification.

Digital image analysis revealed that ACE2 immunostaining in the myocardium of the stage A HF group, visualized with DAB, was similar to that in the control group (Figure 2A). In the stage B HF group, the intensity of ACE2 immunostaining was stronger compared to both the control and stage A HF groups. Notably, ACE2 expression in the stages C/D HF group reached its highest level and was significantly different from the control group, stage A HF group, and stage B HF group. We observed a significant positive correlation between ACE2 immunostaining intensity in myocardial tissue and left ventricular mass (Spearman’s r = 0.58, *p* < 0.001).

### 2.1. ACE2 Expression in Cardiomyocytes

Analysis of cardiomyocyte ACE2 immunostaining revealed that membranous staining of individual cardiomyocytes was the prevalent finding in the control group, with low staining present in more than half of the cases or it being entirely absent (Figure 1A,B). In the stage A HF group, the prevailing observation was moderate ACE2 staining, while in the stage B HF group, more than half of the subjects exhibited very high and high levels of ACE2 staining. In the stages C/D HF group, very high ACE2 immunostaining of cardiomyocytes was prevalent in more than 60% of the participants. In addition to membranous staining, patients in this group also displayed prominent cytoplasmic staining. There was no difference in ACE2 protein expression in cardiomyocytes between patients treated with ACE inhibitors and those not receiving ACE inhibitors (Figure 1C).

The Kruskal–Wallis test with pairwise comparisons was employed to compare immunostained cardiomyocyte scores between each HF group (Figure 2B). The ACE2 immunostaining score in the stage A HF group tended to increase, but there was no significant difference between the stage A HF group and the control group. There was a significant increase in the ACE2 immunostaining score in the stage B HF group compared to the control group (*p* < 0.001). Additionally, there was no difference between the stage B and stage A HF groups. The ACE2 immunostaining score was significantly higher in the stages C/D HF group than in the control group (*p* < 0.001), stage A HF group (*p* < 0.001), and stage B HF group (*p* < 0.05). Notably, there was a significant positive correlation between the ACE2 immunostaining score of cardiomyocytes and the mean DAB intensity of myocardial ACE2 (Spearman’s r = 0.68, *p* < 0.001).

Furthermore, a significant positive correlation was observed between the ACE2 immunostaining score of cardiomyocytes and the mass of the left ventricle (Spearman’s r = 0.60, *p* < 0.001).

### 2.2. ACE2 Expression in Non-Cardiomyocytes

A semi-quantitative assessment of ACE2 immunostaining in non-cardiomyocytes revealed that high levels of ACE2 protein expression were typically detected in the control group, in more than two-thirds of the subjects, as shown in Figure 1B. Conversely, moderate ACE2 immunostaining score was predominantly observed in the stage A and stage B HF groups (in more than three-quarters of the patients). The stages C/D HF group revealed a prevalent low ACE2 immunostaining (in more than 70% of the patients). ACE2 protein expression was similar in patients who took ACE inhibitors and those who did not (see Figure 1C).

The ACE2 immunostaining score demonstrated a significant decrease in the stage A HF group in comparison to the control group (*p* < 0.001, Figure 2C). Similarly, the ACE2 immunostaining score was significantly lower in the stage B HF group when compared to the control group (*p* < 0.001), but no significant difference was observed when comparing the stage B HF group to the stage A HF group. Moreover, in the stages C/D HF group, the ACE2 immunostaining score displayed a significant decline in comparison to the control group (*p* < 0.001), as well as in contrast to both the stage A HF (*p* < 0.001) and stage B HF groups (*p* < 0.001). A significant negative correlation was observed between the ACE2 immunostaining score in non-cardiomyocytes and both the mean DAB intensity of myocardial ACE2 (Spearman’s r = −0.51, *p* < 0.001) and the ACE2 immunostaining score in cardiomyocytes (Spearman’s r = −0.50, *p* < 0.001).

Moreover, a significant negative correlation was observed between the ACE2 immunostaining score in non-cardiomyocytes and the mass of the left ventricle (Spearman’s r = −0.54, *p* < 0.001).

**Figure 3:**
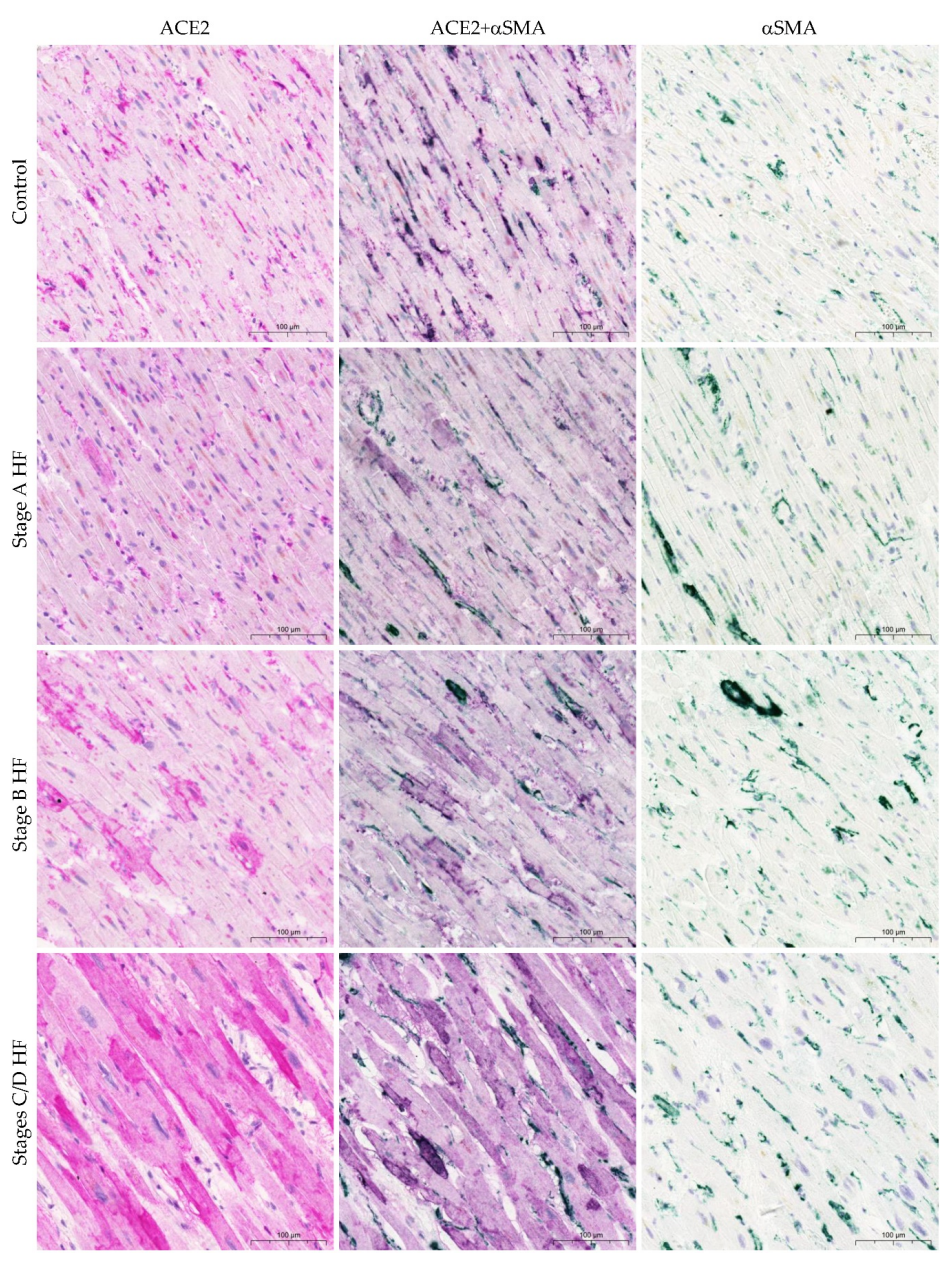
ACE2 protein expression in the human myocardium. Representative images of ACE2 and αSMA co-expression in blood vasculature. Abbreviations: αSMA, alpha smooth muscle actin; ACE2, angiotensin-converting enzyme 2; HF, heart failure; stages A, B, C, and D HF according to ACC/AHA classification. Scale bar: 100 μm.

**Figure 4:**
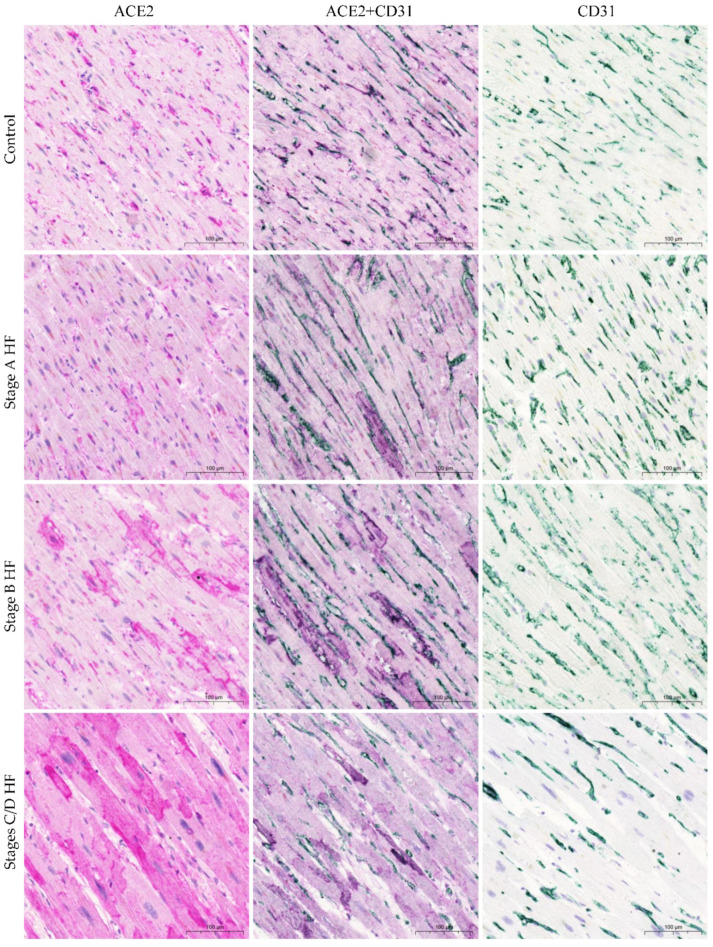
ACE2 protein expression in human myocardium. Representative images of ACE2 and CD31 co-expression in blood vasculature. Abbreviations: ACE2, angiotensin-converting enzyme 2; CD31, cluster of differentiation 31; HF, heart failure; stages A, B, C, and D HF according to ACC/AHA classification. Scale bar: 100 μm.

Finally, ACE2 staining appeared to be localized mainly in cells with thin processes, most of which were associated with the microvasculature and placed adjacent to cardiomyocytes. Based on their morphology and distribution, we hypothesize that these cells showing positive ACE2 expression are pericytes. Double immunohistochemistry for ACE2 and αSMA (as one of the pericyte immunomarkers) unveiled a coincidence between a majority of ACE2 protein expression and αSMA protein expression (Figure 3). Furthermore, we carried out immunohistochemical staining using antibodies that target ACE2 and CD31 (as one of the endothelial cell markers). This confirmed co-expression in a more limited subset of blood vessels (Figure 4).

In conclusion, ACE2 expression is evident in blood vessels, predominantly in pericytes and a small subset of endothelial cells, across all study groups. However, it is more conspicuous in non-diseased myocardial tissue, and its expression diminishes as HF advances.

## 3. Discussion

In this study, we examined the protein expression of ACE2 in 103 samples of left ventricular myocardium tissues using a strategy that employed two independent antibodies, as recommended by the International Working Group for Antibody Validation [27]. Furthermore, one of the antibodies had been previously validated and recommended by the Human Protein Atlas project [18]. The two antibodies used in this study (antibody ab15348, targeting the C-terminal ACE2 domain at 750-805aa, and antibody HPA000288, targeting the N-terminal ACE2 domain at 1-111aa) exhibited consistent staining characteristics in the left ventricular myocardium.

Recent studies have revealed the existence of a short ACE2 isoform, known as delta, in addition to the already recognized long isoform [28]. It is noteworthy that the aforementioned antibodies yielded a similar expression pattern for ACE2 when both ACE2 isoforms were identified (via the ab15348 antibody) and when only the long isoform was identified (via the HPA000288 antibody). Thus, the myocardium predominantly expresses the long isoform of ACE2 in accordance with the results of Blume et al., who observed robust expression of the long ACE2 transcript in the heart using transcript-specific, probe-based qPCR on cDNA, and low expression of the short ACE2 transcript [29]. Moreover, Williams et al. demonstrated that the short isoform of ACE2 protein was not enriched in cardiac tissue through immunohistochemical analysis [30].

To the best of our knowledge, this study represents the first attempt to examine the immunohistochemical representation of ACE2 protein among different myocardial cells corresponding to ischemic heart disease-induced HF stages, namely A (at risk of HF), B (pre-HF), and C/D (symptomatic and advanced HF, respectively) [26]. When ACE2 expression in all myocardial cells was assessed non-selectively by digital image analysis, it remained unchanged in the stage A HF group. However, semi-quantitative analyses of cardiomyocytes showed that their ACE2 membrane protein expression tended to increase, while non-cardiomyocyte ACE2 expression decreased. In the stage B HF group, the intensity of ACE2 immunostaining continued to increase with increasing cardiomyocyte ACE2 expression, whereas non-cardiomyocyte expression was similar to that in the stage A HF group. In the stages C/D HF group, ACE2 protein expression was highest in cardiomyocytes, with both membranous and cytoplasmic staining being prominent. In non-cardiomyocytes, ACE2 expression was lower than in the stage B HF group.

In recent years, several studies have shown that an increase in the activity of the soluble catalytic ACE2 ectodomain in plasma is related to the clinical diagnosis of HF [17]. Additionally, it is associated with a deterioration in New York Heart Association functional class and an increase in B-type natriuretic peptide levels [13,14]. Furthermore, soluble ACE2 activity has been found to be increased in patients with clinical symptoms of HF but who have a preserved left ventricular ejection fraction [13]. Increased soluble ACE2 activity in patients with ST-elevation myocardial infarction has also been found to correlate with infarct size and has been linked to the occurrence of left ventricular remodeling [16]. In patients with angiographically confirmed coronary artery disease, elevated soluble ACE2 activity was found to be a significant predictor of major adverse cardiovascular events [15]. Similarly, plasma ACE2 level was predictive of all-cause and cardiac mortality in the general population [31].

Information on membrane-bound ACE2 is scarce. It has been established that in end-stage HF patients, *ACE2* gene expression in the heart increases in a compensatory manner for left ventricular remodeling [32]. Furthermore, bulk sequencing data have illustrated heightened ACE2 mRNA expression in failing hearts (ischemic cardiomyopathy) as opposed to non-diseased hearts [33]. Recent studies utilizing global quantitative proteomics have revealed that the levels of ACE2 were three times higher, and ACE2 was one of the top 20 proteins that were relatively overexpressed in end-stage HF patients who underwent heart transplantation [24]. Taken together, these studies underscore the necessity of clarifying protein expressions in various cells of the human myocardium through immunohistochemistry.

In the control group of our immunohistochemical study, the most prevalent observation was membranous staining of individual cardiomyocytes. This finding agrees with single-cell sequencing data, which indicated that only 6.6% of cardiomyocytes expressed ACE2 mRNA in healthy hearts [34]. In our study, ACE2 protein expression in left ventricular cardiomyocytes was significantly higher in the stage B HF group (pre-HF)—in other words, before the occurrence of clinical symptoms of HF. At this stage, left ventricular remodeling takes place, potentially leading to higher membrane cardiomyocyte immunostaining [35,36,37]. In the stages C/D HF group, when the remodeling process progressed further, the level of both membranous and cytoplasmic ACE2 protein expression in cardiomyocytes was higher than that of ACE2 expression in the stage B HF group.

Our findings align with those of single-nucleus RNA-Seq studies, where higher cardiomyocyte *ACE2* gene expression was found in failing hearts compared to non-diseased hearts. However, it is worth noting that these trials encompass patients with HF not related to ischemic heart disease [21,23]. In the immunofluorescence study conducted by Vukusic et al., it was found that patients with different causes of HF exhibited an elevated expression of ACE2 protein in cardiomyocytes when compared to control subjects [24]. Bargehr et al. investigated ACE2 protein expression in patients who underwent heart transplantation due to ischemic heart disease-induced HF [25]. They compared this group to other groups with different causes of HF, but they did not have a control group. Our study revealed that ACE2 expression in the myocardium (overall) and cardiomyocytes began to increase during the pre-symptomatic stage of heart failure (stage B HF group) and continued to rise with advancing HF. The fact that ACE2 expression was associated with left ventricular mass suggests that these changes in ACE2 expression were linked to the process of left ventricular remodeling. The initial study on the function of ACE2 as a vital regulator of heart function surfaced promptly after its revelation [38]. ACE2 cleaves angiotensin II to produce angiotensin 1-7, and it cleaves angiotensin I to generate angiotensin 1-9. The primary mode of operation of these compounds is believed to be associated with their binding to the MasR and angiotensin II type 2 receptor (AT_2_R), respectively [39]. Signaling through the AT_2_R can directly inhibit AT_1_R activation, thereby obstructing the effects of angiotensin II [40]. Activation of the AT_2_R can reduce cardiomyocyte hypertrophy by inhibiting extracellular signal-regulated kinase 1 (ERK1) and ERK2 through the activation of Src homology region 2 domain-containing phosphatase 1 (SHP1) and mitogen-activated protein kinase-phosphatase 1 [41,42]. Additionally, the activation of AT_2_R via angiotensin 1-9 binding has been linked to cardioprotective effects through Akt phosphorylation [43]. Angiotensin 1-7 induces the nitric oxide (NO)-soluble guanylyl cyclase pathway, leading to vasodilation through the activation of the proto-oncogene MasR. The activation of this receptor can also reduce cardiac fibrosis by stimulating SHP1 and dual-specificity phosphatase, thereby inhibiting p38 mitogen-activated protein kinase and ERK1 and ERK2 [44,45,46]. Furthermore, angiotensin 1-7 has an anti-hypertrophic impact by obstructing the nuclear factor of activated T cells through a MasR-phosphoinositide 3-kinase-Akt-NO-cyclic guanosine monophosphate-dependent pathway [47].

Examining ACE2 expression in non-cardiomyocytes highlights a distinct range of alterations. Early research into ACE2 discovered that the ACE2 protein is situated in cardiac endothelial cells and smooth muscle cells through immunohistochemistry [48]. Furthermore, recent analysis through single-nucleus transcriptomics revealed that *ACE2* expression was present in non-diseased hearts, including endothelial cells, fibroblasts, and pericytes, in addition to cardiomyocytes. Unexpectedly, pericytes exhibited higher levels of *ACE2* expression than cardiomyocytes [22,23]. He et al. noted that not all cardiac capillary pericytes were strongly ACE2 positive; some were weakly positive, and some were negative [49]. We found that ACE2 protein expression was highest in non-cardiomyocytes in the myocardium of the control group patients. Non-cardiomyocyte ACE2 immunoreactivity was lower in both stage A (at risk of HF) and stage B (pre-HF) HF groups, compared to the control group. In the non-cardiomyocytes of stages C/D HF group, ACE2 protein expression was lowest. Taking into account non-cardiomyocyte cell morphology, their location, and the antigenic profile marker (αSMA-positive cells) [50], we observed that the pericytes exhibited the most notable expression of ACE2 protein, with a smaller proportion of ACE2-immunopositive cells coming from the endothelial cells (CD31-positive cells). A previous immunohistochemical study of the heart has suggested that myocardial ACE2 protein expression is lower in HF patients in pericytes, endothelial cells, and fibroblasts, as compared to cardiomyocytes [25]. However, no comparisons with non-diseased myocardium were made. Our study of the stages C/D HF group supports transcriptomic investigations that demonstrate lower *ACE2* expression in pericytes and fibroblasts of HF patients compared to non-failing myocardium [23]. The factors that determine these divergent trends in ACE2 protein expression in cardiomyocytes and non-cardiomyocytes are unclear. However, it is most likely associated with myocardial remodeling characteristics, where the capillary network becomes inadequate during the hypertrophy of cardiomyocytes [51,52]. This could be supported by a significant negative correlation between the ACE2 immunostaining score in non-cardiomyocytes and the left ventricular mass documented in our study.

In addition, our data are in accordance with the finding of studies that reported no dysregulation of ACE2 bound to the membrane with pharmacological inhibition of angiotensin-converting enzyme [23,25].

The limitations of this study must be acknowledged. Archival formalin-fixed and paraffin-embedded tissues prove difficult to stain through immunofluorescence due to auto-fluorescence related to the fixation process. Hence, our assessment can only be qualitative for the co-localization of ACE2 and various cell markers, such as αSMA (a pericyte marker) and CD31 (an endothelial cell marker).

## 4. Materials and Methods

### 4.1. Study Design and Groups

Myocardial tissue samples from the middle segment of the human left heart ventricle were selected from the paraffin blocks archive of the Laboratory of Cardiac Pathology of the Institute of Cardiology (Lithuanian University of Health Sciences). Selected myocardial tissue samples were further classified into three groups by the stages of HF based on the American College of Cardiology (ACC)/American Heart Association (AHA) classification [26].

The demographic and clinical characteristics of the study groups are shown in Table 1. The stage A HF (at risk for HF) group was comprised of previously healthy individuals or whose state of health had improved or stabilized prior to death and who died suddenly due to ischemic heart disease within six hours of experiencing symptoms [53,54]. The patients of this group had no previous symptoms of HF. A complete morphological investigation of the heart was performed during postmortem examinations, and no scars after myocardial infarction were revealed and acute ischemic injuries were within the period of six hours [55]. The stage B HF (pre-HF) group was defined by the same characteristics as the previous group, but old myocardial infarctions (scars) in the postmortem morphological investigation were detected. The stages C/D HF (symptomatic and advanced HF) group consisted of patients who underwent a heart transplantation procedure, and an extensive morphological examination of surgical material was carried out.

The control group comprised patients who died from either external causes or acute non-cardiovascular diseases, whose hearts were examined postmortem.

All patients in the study groups did not have any prior diagnoses of conditions, such as systemic arterial hypertension, congenital or acquired cardiac valve disease, cardiomyopathy, diabetes mellitus, or pulmonary diseases that could lead to heart remodeling. Before immunohistochemical analysis, all selected cases underwent a comprehensive histological examination. Therefore, myocardial tissue samples were carefully extracted with special attention to avoiding the areas affected by acute ischemic injury or post-myocardial infarction scarring.

The study was conducted in accordance with the Declaration of Helsinki and approved by Kaunas Regional Biomedical Research Ethics Committee (No. BE-2-77, 2022).

### 4.2. Immunohistochemistry

Formalin-fixed and paraffin-embedded tissue blocks from the left ventricle were cut into 3 µm slices using a Leica RM2235 microtome (Leica Biosystems, Deer Park, IL, USA) and then mounted on Menzel SuperFrost Plus slides (Menzel, Braunschweig, Germany). Subsequently, the slides were allowed to air-dry at room temperature overnight and were then baked at 50 °C for a minimum of 12 h. The tissue sections underwent deparaffinization with xylene, followed by sequential immersion in decreasing concentrations of ethyl alcohol and rehydration with distilled water.

Antigen retrieval was carried out using the RHS-1 microwave tissue processor (Milestone Medical, Bergamo, Italy). The samples were exposed to TRIS/EDTA buffer (pH 9.0) (Agilent Technologies Inc., Wood Dale, IL, USA, S236784-2) and incubated at 110 °C for 8 min. For the immunohistochemistry process, Sequenza slide racks and cover plates (Shandon Diagnostics Limited, Runcorn, UK) were employed. The tissue sections were treated with peroxidase-blocking solution (Agilent Technologies Inc., Wood Dale, IL, USA, S202386-2) for 15 min and were subsequently rinsed with wash buffer (Agilent Technologies Inc., Wood Dale, IL, USA, S300685-2).

**Figure 5:**
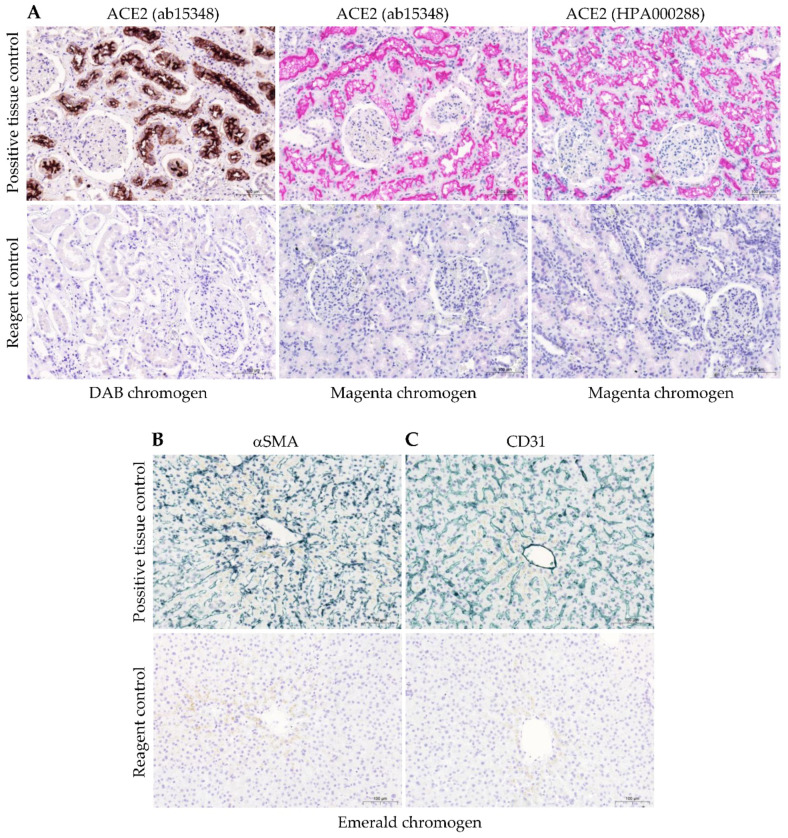
Positive tissue and negative reagent controls for ACE2 which were used for both antibodies and different chromogens ((**A**) kidney: moderate to strong ACE2 staining was detected in the tubular cells without glomerular expression); for αSMA ((**B**) liver: the majority of the perisinusoidal cells showed a weak-to-moderate, distinct cytoplasmic staining reaction); for CD31 ((**C**) liver: all endothelial cells of the sinusoids exhibited a weak-to-moderate predominantly membranous staining reaction). Abbreviations: αSMA, alpha smooth muscle actin; ACE2, angiotensin-converting enzyme 2; CD31, cluster of differentiation 31. Scale bar: 100 μm.

Two distinct antibodies were used to assess ACE2: polyclonal rabbit anti-Human ACE2 Ab15348, which targets the 750-805aa epitope (Abcam, Cambridge, UK), and polyclonal rabbit anti-Human ACE2 HPA000288 antibody (Sigma Aldrich, Merck Group, St. Louis, MO, USA), recognizing the 1-111aa epitope, in accordance with recommendations by the International Working Group for Antibody Validation [27]. Primary antibodies were appropriately diluted with antibody diluent (Agilent Technologies Inc., Wood Dale, IL, USA, S080983-2), as specified in Table 2, and were incubated at room temperature for 60 min. The sections were processed using the EnVision FLEX visualization system with rabbit linker and HRP DAB+ or HRP magenta chromogen (Agilent Technologies Inc., Wood Dale, IL, USA, K800221-2, K800921-2, GV92511-2), following the manufacturer’s instructions. Hematoxylin counterstaining (Agilent Technologies Inc., Wood Dale, IL, USA, S330930-2) was performed, followed by a dehydration process with increasing concentrations of ethyl alcohol and xylene before permanent coverslipping. Both primary antibodies targeting anti-human ACE2 were used for positive control staining in human kidney sections (3 µm in thickness). This step was undertaken to ensure the specificity of the primary antibodies (Figure 5A). The primary antibodies were also tested against the corresponding isotype control, which did not yield any specific staining.

For the purpose of determining protein co-localization, tissue sections from the left ventricle were subjected to double immunohistochemistry staining for ACE2-CD31 and ACE2-alpha smooth muscle actin (αSMA). Initially, these sections were incubated with polyclonal anti-human ACE2 (ab15348) antibodies derived from rabbits after undergoing an antigen retrieval process and enzyme blocking, as previously described. Subsequently, the ACE2 protein was visualized using HRP magenta chromogen, with an endogenous enzyme block being performed using 0.3 M sulfuric acid. This process was replicated for αSMA (Agilent Technologies Inc., Wood Dale, IL, USA, M085101-2) or CD31 (Agilent Technologies Inc., Wood Dale, IL, USA, M082301-2). Hematoxylin counterstaining was conducted before applying the emerald chromogen (Neo-Biotech, Nanterre, France, NB-23-00153) in accordance with the manufacturer’s instructions. Three serial slides were stained: the first slide was reserved for ACE2 alone, the second slide underwent double staining for ACE2 and αSMA (or CD31), while the third slide was designated for αSMA (or CD31) alone. Liver tissue was employed as a positive control for both αSMA (Figure 5B) and CD31 (Figure 5C) by following the same immunohistochemical reaction protocol used for the analyzed myocardial tissue samples. The reactions were conducted simultaneously, following the guidelines published by the International Ad Hoc Expert Committee [56]. As a reagent control, IgG of the same isotype class was used at the same dilution as the primary antibody.

### 4.3. Image Acquisition and Analysis

The stained slides were digitally scanned using a Pannoramic MIDI (3DHistech, Budapest, Hungary) equipped with a 20×/0.8 objective lens and a Hitachi HV-F22CL camera, providing an image resolution of 0.242 µm per pixel.

Whole-slide images of the myocardium, where ACE2 was visualized using the DAB chromogen, were analyzed using QuPath v0.4.3 [57]. The images were imported into the software and labeled with their corresponding names to prevent biased selection. In each slide, 15 randomly chosen annotation areas, each measuring 235,858 µm^2^, were designated for analysis. Mean DAB intensity measurements were conducted to assess ACE2 expression in each of the selected annotations.

Semi-quantitative assessment was carried out using SlideViewer (3DHistech, Budapest, Hungary). Two independent investigators manually annotated all tissue samples where ACE2 was visualized by magenta chromogen staining. Annotation parameters were established using a 5-grade scale for cardiomyocytes, considering staining intensity and the quantity of stained cells. These parameters were based on the workflow outlined in the Human Protein Atlas [18]: 0 = not detected (negative or weak staining in less than 25% of cells); 1 = low (weak staining in at least 25% of cells or moderate staining in less than 25% of cells); 2 = medium (moderate staining in at least 25% of cells or strong staining in less than 25% of cells); 3 = high (strong staining of at least 25% but less than 50% of cells); 4 = very high (strong staining of at least 50% of cells). For the quantification of immunohistochemical staining in non-cardiomyocyte cells, a 4-grade scale was employed: 0 = not detected (negative or weak staining in less than 25% of cells); 1 = low (weak staining in at least 25% of cells or moderate staining in less than 25% of cells); 2 = medium (moderate staining in at least 25% of cells or strong staining in less than 25% of cells); 3 = high (strong staining of at least 25% of cells).

The consensus classification was determined by considering all individual samples, and any challenging cases were discussed with a certified pathologist (D.P.), who acted as an independent observer. All images were anonymized, and primary measurements were conducted in a blinded manner by the observer. To validate the findings, a representative sample of images was analyzed by an independent investigator in a blinded fashion to ensure the reproducibility of the measurements.

### 4.4. Statistical Analysis

The normality of the distribution of continuous variables was assessed using the Shapiro–Wilcoxon test. Continuous variables were expressed as either means with standard deviation (or standard error in nested ANOVA) or medians with interquartile ranges, as deemed appropriate. Differences between groups were analyzed using nested ANOVA with post hoc Bonferroni tests for the multiple comparisons and a Kruskal–Wallis test with pairwise comparisons adjusted according to Bonferroni’s method. Spearman’s rank correlation test was employed to evaluate correlation trends. Statistical significance was determined at a threshold of *p* < 0.05. The statistical analysis was conducted using the Statistical Package for the Social Sciences (SPSS) software (SPSS Statistics Version 29.0, IBM, Armonk, NY, USA).

## 5. Conclusions

In the presented study, it was shown that alterations in ACE2 protein expression began to occur at the early stage of ischemic HF, specifically in stage A HF, during which individuals are at risk for HF. These modifications advanced in stage B HF, which is pre-HF, and also in stages C/D HF, which refers to symptomatic and advanced HF. The changes take place differently in diverse myocardial cells. ACE2 membrane protein expression in cardiomyocytes showed a tendency to increase in the stage A HF group, while there was a significant rise in the intensity of ACE2 immunostaining in the stage B HF group. The most notable expression of ACE2 was found in the stages C/D HF group. In non-cardiomyocytes, ACE2 protein expression was significantly lower in the stage A HF group than the control group, but there was no significant difference in expression between the stage B and A HF groups. In the stages C/D HF group, ACE2 expression in non-cardiomyocytes was at its lowest. These changes in ACE2 protein levels are associated with left ventricular remodeling in ischemic HF. The novelty of our study is that we investigated alterations in ACE2 protein expression not only in the symptomatic and advanced stages of ischemic HF, but also in the pre-symptomatic stages. These findings, obtained via immunohistochemical techniques, enhance our comprehension of ACE2 protein expression in the human heart. They offer noteworthy verification sources for ongoing sequencing research.

## Figures and Tables

**Table 1:** Characteristics of the study population by groups.

Characteristic	Control Group *n* = 21	Stage A HF Group *n* = 25	Stage B HF Group *n* = 23	Stages C/D HF Group *n* = 34
Age, mean (SD), years	50.2 (8.1)	53.8 (8.0)	54.4 (7.7)	56.2 (7.2)
Sex	Male	Male	Male	Male
Previous clinical symptoms of HF	No	No	No	Yes
Treatment with ACE inhibitors (ramipril, perindopril)	No	No	No	Yes (23)/No (11)
Treatment with angiotensin receptor blockers	No	No	No	No
Atherosclerotic stenosis ≥ 75% in at least one coronary artery	No	Yes	Yes	Yes
Old myocardial infarction	No	No	Yes	Yes
Mass of left ventricular free wall without subepicardial adipose tissue, mean (SD), g	104.0 (11.9)	133.4 (21.0)	155.0 (33.6)	193.6 (26.7)

ACE, angiotensin-converting enzyme; HF, heart failure; stages A, B, C, and D HF according to the ACC/AHA classification; SD, standard deviation.

**Table 2:** Characteristic of primary antibodies used for immunohistochemistry.

Antibody	Species (Immunogen)	Dilution	Manufacturer (Catalog Number)	PRID	Lot Number
ACE2	Rabbit polyclonal (Synthetic peptide within human ACE2 aa 750 to the C-terminus)	1:500	Abcam (ab15348)	AB_301861	GR3410089-1
ACE2	Rabbit polyclonal (angiotensin-converting enzyme 2 precursor recombinant protein epitope signature tag (PrEST) 1-111aa)	1:500	Sigma Aldrich (HPA000288)	AB_1078160	000013837
αSMA	Mouse monoclonal, cl 1A4 (N-terminal synthetic decapeptide of αSMA)	1:100	Agilent Dako (M085101-2)	AB_2223500	41480058
CD31	Mouse monoclonal, cl JC70A (the epitope recognized was found to be within the extracellular domain 1)	1:100	Agilent Dako (M082301-2)	AB_2114471	41495396

Abbreviations: ACE2, angiotensin-converting enzyme 2; αSMA, alpha smooth muscle actin; CD31, cluster of differentiation 31.

## Data Availability

The data that support the findings of this study are available from the corresponding author upon request.

## Abbreviations

ACC/AHA: American College of Cardiology/American Heart Association; ACE: angiotensin-converting enzyme; ACE2: angiotensin-converting enzyme 2; ACEi: angiotensin-converting enzyme inhibitors; αSMA: alpha smooth muscle actin; AT_1_R: angiotensin II type 1 receptor; AT_2_R: angiotensin II type 2 receptor; CD31: cluster of differentiation 31; DAB: diaminobenzidine; HRP: horseradish peroxidase; ERK1: extracellular signal-regulated kinase 1; ERK2: extracellular signal-regulated kinase 2; HF: heart failure; MasR: Mas receptor; NO: nitric oxide; SD: standard deviation; SHP1: Src homology region 2 domain-containing phosphatase 1.
